# Male partners involvement in human immune deficiency virus testing and counseling during prenatal care visits in Bichena town Westcentral Ethiopia: a cross-sectional study

**DOI:** 10.1186/s13104-022-06215-9

**Published:** 2022-10-20

**Authors:** Nigusu Ayalew Gessesse, Getahun Belay Gela, Amlaku Mulat Aweke, Wondu Feyisa Balcha

**Affiliations:** Department of Midwifery, College of Medicine and Health Sciences, Bahir Dar University, P.Box:079, Bahir Dar, Ethiopia

## Abstract

**Objective:**

Transmission of the Human immune deficiency virus (HIV) from mother to child remains a significant problem in developing countries. Male partners’ involvement in HIV testing and counseling is a major entry point for the prevention of mother-to-child transmission (PMTCT) of HIV. This study aimed to assess male partners’ involvement in HIV testing and counseling during prenatal care visits in Bichena town, Westcentral Ethiopia.

**Results:**

A community-based cross-sectional study design was conducted from October 1/2018 to June 15/2019 among 406 male partners. Less than half (41.38%) [95% CI: 36.20–46.10%] of the male partners were involved in HIV testing and counseling. In multivariable analysis male partners who are found in the age group of 20–29 years, secondary, and diploma and above educational level, good knowledge of the services provided in the prenatal care visits, male partners whose wife had ≥ 4 prenatal care visits, good knowledge of mother to child transmission (MTCT) and PMTCT of HIV, entering the prenatal care room together with his wife, discussing maternal health issue with health care providers, and travel < 15 min to reach a nearby health facility were associated with male partners involvement in HIV testing and counseling.

**Supplementary Information:**

The online version contains supplementary material available at 10.1186/s13104-022-06215-9.

## Introduction

Human Immunodeficiency Virus (HIV) counseling and testing is the access point to HIV prevention, care, and treatment programs [1]. The World Health Organization (WHO) recommends offering HIV testing and counseling (HTC) to couples, wherever HTC is available, including in prenatal care/PMTCT clinics [2]. In Ethiopia, the HIV testing algorithm includes three rapid test kits (RTKs): STAT PACK (screening test), ABON (confirmatory test), and SDBIOLINE (tie-breaker test) [3]. Deoxyribonucleic Acid Polymerase Chain Reaction (DNA/PCR) test is also used for children under the age of 18 months [4]. The WHO also recommends testing for hepatitis B virus (HBV) surface antigen (HBsAg) 1 and syphilis at least once and as early as possible in the pregnancy [5]. Due to similar blood-born transmission routes of HBV and hepatitis C virus (HCV) infections are common among HIV-positive individuals [6–8].

Without intervention, the risk of HIV infection from infected pregnant mothers to their unborn children ranges from 25 to 35% in developing countries, while with the implementation of core PMTCT interventions, this risk can be reduced to 2–5% [9]. In Ethiopia, the prevalence of MTCT of HIV ranges from 4.16 to 15.7% [10, 11], with a pooled prevalence of 9.93% [12]. Male partner involvement (MPI) in HTC increases the use of PMTCT interventions and improved HIV-free survival among infants [13–16]. However, the low MPI in the PMTCT results in pregnant women not getting tested for HIV [17]. A study conducted in Uganda indicates that more than 50% of pregnant women who refused HTC in a PMTCT setting reported the need for male partner (MP) permission before they could be tested [18].

In Ethiopia, the rate of MTCT of HIV is still > 5% since the implementation of the option B + regime [19]. Lack of support and absence of MPI is the main reason for the low option B + regimen adherence [20]. However, HIV testing and counseling of pregnant women with their MP is essential to initiate early antiretroviral therapy that improves maternal health and decreases the risk of MTCT [21]. MPI in HTC is critical for PMTCT [22], and it has been recognized as an apriority focus area to be strengthened in PMTCT [23, 24]. Ending the epidemics of HIV/AIDS and eliminating MTCT of HIV is a global public health priority [25]. In Ethiopia, despite the availability of antiretroviral treatment, MTCT of HIV is still higher [26]. Therefore, assessing the level of MPI in HTC during prenatal care visits, and its associated factors are important for the implementation of the Sustainable Development Goals [27].

## Materials and methods

### Study design and period

A community-based cross-sectional study was conducted from October 1/2018 to June 15/2019 in Bichena town, Westcentral Ethiopia.

### Study setting

This study was conducted in Bichena town, which is located in the East Gojjam Zone of the Amhara Region, Ethiopia. The town has five kebeles (the lowest administrative unit in Ethiopia, next to the district) with a total number of households of 7174 [28].

### Study participants

All MP whose wives gave birth within the last one year before the study and had prenatal care visits in Bichena town.

## Operational definitions

### Male partner

an individual with whom the pregnant woman was in an intimate sexual relationship, and was responsible for her pregnancy whether they were legally married or not [29].

### Male partner involvement in HTC

Refer to the MPI in both HIV counseling and testing during his wife’s prenatal care visit [29].

### Knowledge of MTCT

When MP knows at least one timing of transmission of MTCT of HIV from three questions (during pregnancy, delivery, and breastfeeding), considered as having knowledge of MTCT [29, 30].

### Knowledge of PMTCT

When MP knows at least one PMTCT method from the three knowledge measuring items (using anti-retroviral therapy, delivery by cesarean section, and avoiding breastfeeding), considered to as having knowledge of PMTCT [29, 30].

### Sample size determination

The sample size was calculated using a single population proportion formula by considering the following assumptions: the proportion of MPI in HTC at Gondar town was (40.10%) [29], Zα/2 = critical value for normal distribution at 95% confidence level, which is equal to 1.96 (Z value of alpha = 0.05) or 5% level of significance (α = 0.05) and a 5% margin of error (d = 0.05). The sample size was adjusted by adding a 10% non-response rate and the final sample size was 406.

### Sampling procedure and techniques

The total sample size was proportionally allocated for each kebele of the town based on the total number of MP whose wives gave birth within the last one year and had prenatal care visits. Before data collection, a census was conducted to identify MP whose wives gave birth within the last one year and had prenatal care visits in each kebele of the town. The total number of MP whose wives gave birth within the last one year and had prenatal care visits in the five kebeles of the town was 864. The study participants were selected by simple random sampling techniques.

### Data collection tools and procedures

The questionnaire was developed by the authors after reviewing different kinds of literature on the topic [29–33]. The data were collected using structured and pre-tested interviewer-administered questionnaires through face-to-face interviews. The data was collected by five-diploma midwives and supervised by two BSc midwives.

### Data quality control

The data collectors and supervisors trained for two days on the objectives, relevance of the study, and techniques of interviews. The questionnaire was pre-tested before the actual data collection period on 5% (20) MP at Debre Werke town to ensure the clarity of the questionnaire and confirm the logical sequence of the questions.

### Data processing and analysis

The data were entered by using Epi data 3.1, then exported to SPSS version 23.0 for analysis. All explanatory variables which have a significant association in bivariate analysis with a P-value < 0.20 were entered into a multivariable logistic regression model to get an adjusted odds ratio (AOR), and those variables with 95% of confidence intervals (CI) and a P-value of < 0.05 was considered as statistically significant with MPI in HTC.

## Results

### Socio-demographic characteristics of the male partners

A total of 406 MP participated with a response rate of 100%. The mean age of the MP was 35.08 years and ranged from 20 to 60 years. Of the MP, 124 (30.54%) had a diploma and above educational level (Table 1).

**Table 1:** Socio-demographic characteristic of the MP in Bichena town, Westcentral, Ethiopia, 2019, (n = 406)

Variables	Categories	Frequency	Percentage
Age of male partners	20–29 30–39 40–60	88 216 102	21.68 53.20 25.12
Religion	Orthodox Muslim Protestant	327 68 11	80.54 16.75 2.71
Male partners’ education level	Had no formal education Primary school Secondary school Diploma and above	89 109 84 124	21.92 26.85 20.69 30.54
Male partners occupation	Employed (public/NGO) Farmer Merchant Driver Others*	95 139 89 39 44	23.40 34.23 21.92 9.61 10.84
Wives education level	Had no formal education Primary school Secondary school Diploma and above	110 114 94 88	27.09 28.08 23.15 21.68
wives occupation	Housewife Employed (public/NGO) Merchants Others**	235 68 72 31	57.88 16.75 17.73 7.64
Age of male partners at first marriage	15–19 20–24 25–29 ≥ 30	81 144 137 44	19.95 35.47 33.74 10.84
Length of marriage	≤ 10 11–20 ≥ 21	143 187 76	35.22 46.06 18.72
Family size	3–4 ≥ 5	214 192	52.71 47.29
Number of under-five children	One child Two or more children	299 107	73.64 26.36
Is the pregnancy was planned	Yes No	384 22	94.58 5.42

* Driver, daily laborer, and carpenter, ** student, and daily laborer

### Knowledge of male partners about the services provided in prenatal care/PMTCT clinic

Two third (65.77%) of the MP had good knowledge of the services in prenatal care clinics. The availability of HTC, and PMTCT knew by 249 (61.33%), and 177 (43.60%) of MP respectively. Nearly four in ten (41.38%) of MP knew the provision of hepatitis virus screening during prenatal care visits (Supplementary material 1 Table S1).

### Involvement in prenatal care/PMTCT

About, 287 (70.70%) MP was involved in making a joint prior plan for the prenatal care visit. Of the MP, 214 (52.70%) accompanied their wife to prenatal care visit at least once, and 196 (48.30%) physically entered the prenatal care/PMTCT room together with their wives (Figure S1).

### Health care facility and cultural influence

Of the MP, 244 (60.10%) have a health facility nearby to their home, which is taking about 15–30 min to reach, and 393 (96.80%) knew that the maternal health service provision at public health facilities is free (Supplementary material 1 Table S2).

### Level of male partners’ involvement in HTC

In this study, 168 (41.38%) with [95% CI = 36.20–46.10) of MP involved in HTC. Of the MP, 249 (61.33%), and 177 (43.60%) knew at least one timing of MTCT, and PMTCT methods respectively (Table 2).

**Table 2:** Level of MPI in HTC in Bichena town, Westcentral, Ethiopia, 2019, (n = 406)

Variables	Categories	Frequency	Percentage
Visited the PMTCT/prenatal care clinic with his wife at least once	Yes No	214 192	52.71 47.29
Reasons for not accompanying to the PMTCT/prenatal care clinic, (n = 192)*	The inflexibility of PMTCT/prenatal care visiting time Work overload Long waiting time Prenatal care/PMTCT is a concern of the women Limited space to accommodate both parents Other family members accompanied her Male feel shame Women do not allow their partners to accompany Do not know pregnancy could result in a complication	165 145 139 88 66 103 45 37 31	85.94 75.52 72.39 45.83 34.38 53.65 23.44 19.27 16.14
Number of male partners in prenatal care visited (n = 214)	1 2 3 ≥ 4^+^	125 49 26 14	58.41 22.89 12.15 6.54
Numbers of wives prenatal care visited	< 3 ≥ 4^+^	126 280	31.03 69.97
Involved in both testing and counseling of HIV	Yes No	168 238	41.38 58.62
Know at least one timing of MTCT	Yes No	249 157	61.33 38.67
Timing of MTCT (n = 249)*	During pregnancy During delivery During breastfeeding	146 187 74	58.63 75.10 29.72
Knew at least one PMTCT method	Yes No	177 229	43.60 54.40
Methods of PMTCT (n = 177)*	Anti-retroviral therapy Delivery by cesarean section Avoiding breastfeeding	158 29 43	89.27 16.38 24.29

* Multiple responses are possible

### Factors associated with MPI in HTC

In bivariate analysis variables with a P-value of less than 0.20 were entered into the multivariable analysis. In the multivariable analysis: MP`s who are found in the ages group of 20–29 years, secondary, and diploma and above educational level, having good knowledge of the services provided in prenatal care visits, knowing at least one timing of MTCT and PMTCT method, entering the prenatal care/PMTCT clinic together with his wife, discussed maternal health issues with health care providers, whose wife had four and above prenatal care visits, and traveled < 15 min to reach a nearby health facility were significantly associated with MPI in HTC at a P-value of less than 0.05 (Table 3).

**Table 3:** Logistic regression analysis for the MPI in HTC at Bichena town, Westcentral, Ethiopia, 2019, (n = 406)

Variables	Involved in HTC		COR( 95% CI)	AOR(95% CI)	P-value
	**Yes**	**No**			
Age of male partners					
≥ 40 30–39 20–29	23 81 64	79 135 24	1 2.06 (1.20–3.54) 9.16 (4.73–17.72)	1 1.59 (0.61–4.17) **4.05 (1.23-113.34)**	0.345 0.021*
Male partners’ education level					
Had no formal education Primary education Secondary education Diploma and above	11 37 38 82	78 72 46 42	1 3.64 (1.73–7.68) 5.85 (2.73–12.57) 13.84 (6.65–28.80)	1 1.08 (0.31–3.74) **4.13 (1.09–15.67)** **5.32 (1.55–18.26)**	0.909 0.037* 0.008*
Wives education level					
Had no formal education Primary education Secondary education Diploma and above	40 65 40 43	70 69 54 54	1 1.14 (0.66–1.96) 1.30 (0.74–2.29) 1.67 (0.95–2.96)	1 0.56 (0.18–1.75) 0.65 (0.21–2.01) 0.53 (0.15–1.87)	0.316 0.455 0.326
Knowledge of male partners about prenatal care					
Poor knowledge Good knowledge	19 149	120 118	1 7.95 (4.64–13.70)	1 **3.25 (1.13–9.35)**	0.029*
Knew at least one timing of MTCT					
No Yes	39 129	118 120	1 3.25 (2.10–5.05)	**3.17 (1.34–7.49)**	0.009*
Knew at least one PMTCT method					
No Yes	56 112	173 65	1 5.32 (3.47–8.18)	1 **2.55 (1.10–5.91)**	0.030*
Physically entered the prenatal care/PMTCT clinic together with his wife					
No Yes	18 150	192 46	1 34.78 (19.37–62.45)	1 **15.77 (6.48–38.35)**	0.001*
Numbers of wives prenatal care visited					
< 3 times 4^+^	20 148	106 132	1 5.94 (3.49–10.12)	1 **3.13 (1.17–8.38)**	0.023*
Discussed maternal health issues with health care providers					
No Yes	52 116	219 19	1 25.71 (14.52–45.54)	1 **14.65 (6.44–33.31)**	0.001*
Time to reach the nearest health facility					
> 30 min 15–30 min ≤ 15 min	28 100 40	76 144 18	1 1.88 (1.14–3.12) 6.03 (2.98–12.21)	1 0.81 (0.31–2.13) **3.99 (1.11–14.31)**	0.667 0.034*
Decision-maker to seek health facility					
Ether husband/wife Together	8 160	43 195	1 4.41 (2.01–9.65)	1 1.27 (0.34–4.76)	0.720

*Significant at a P-value of < 0.05

## Discussion

Only screening pregnant mothers are not satisfactory for PMTCT, it is necessary involving MP to improve women’s uptake of PMTCT services [34, 35]. This study identified 41.38% [95% CI: 36.20–46.10%] of the MP involved in HTC. This finding is in line with a study conducted in Gondar Town (40.10%) [29]. However, the finding of this study is higher than studies conducted in Goba town (22.70%), and Mekelle town (16.50%) [35, 36]. The implementation of health extension workers increases the awareness of MP on the advantage of involving in HTC [37, 38]. The finding of this study is lower than studies conducted in Delanta District (53.70%) [31], Arba Minch (53.60%) [33], Debre Markos town (59.10%) [39], and Addis Ababa (63.70%) [32]. The discrepancy might be the differences in the study setting, and the provision of health care services differs from facility to facility.

Male partners who are found in the age group of 20–29 years were 3.34 times more likely involved in HTC. It is in line with other studies [29, 36]. Young age group MP may pay more care and attention to their wife. MPI in HTC is increased with increasing educational levels. This finding is in line with other studies [15, 33, 40]. Educated men have more information regarding the advantage of getting the service provided at the health facility.

Entering the PMTCT clinic together with wives increased the MPI in HTC by 15.77 times. It is in line with another study [31]. During the counseling session, they may understand that HTC is the responsibility of both parents. The odds of MPI in HTC were 3.13 times higher among MP whose wives had ≥ 4 prenatal care visits. This is agreed with another study [31]. Having support from the husband makes the pregnant woman to completion of the prenatal care visits, which in turn also increases MPI in HTC [41]. MP`s who discussed maternal health issues with health care providers were 14.65 times more likely involved in HTC. Making a bilateral decision regarding the health of families increases the utilization of health care services [42–44].

Having a health facility nearby to the living home increased MPI in HTC. This is congruent with another study [33]. This might be making the visiting time convenient. Knowing the service provided at the PMTCT clinic increased MPI by 3.25 times. This finding is in line with other studies [15, 33, 45]. The MPI in HTC was 3.17 times higher among MP who knew at least one timing of MTCT. This is consistent with other studies [29, 32, 46]. MP who know at least one PMTCT method were 2.55 times more likely involved in HTC. This finding agrees with other studies [15, 32, 33, 39]. Knowing MTCT/PMTCT could increase the MPI in HTC and it may also make them understand that pregnancy is their shared responsibility.

Less than half (41.38%) of the MP mentioned screening for hepatitis virus as one of the services provided in the prenatal care clinics. Hepatitis virus infection is a public health problem among pregnant women and the risk of acquiring HBV infection in HIV-infected patients is higher compared to HIV-negative individuals [47]. Besides the HTC of the MP, screening the MP for hepatitis virus and creating awareness is a good entry point to prevent its complications [48].

## Conclusion

Male partners’ involvement in HTC in the study area was low compared to other studies conducted in Ethiopia. We suggest emphasizing awareness creation on the timing of MTCT of HIV, PMTCT methods, and the services provided at prenatal care visits. The health care providers should have to encourage the MP to have HTC during his wife’s pregnancy and to discuss maternal health issues. Increasing access to education, encouraging women to have at least four prenatal care visits, making health facilities accessible, and designing programs that will encourage MP involvement in HTC are important.

## Limitation

This study has certain limitations: it was not triangulated with the qualitative method. Recall bias might be introduced on some of the questions that required the MP to recall past information.

## Electronic supplementary material

Below is the link to the electronic supplementary material.

Supplementary Material 1

## Data Availability

All related data have been presented within the manuscript. The data set supporting the conclusion of this article is available from the corresponding author upon reasonable request.

## Abbreviations

AIDS: Acquired Immune Deficiency Syndromes.
AOR: Adjusted Odds Ratio.
CI: Confidence Interval.
COR: Crude Odds Ratio.
DNA/PCR: Deoxyribonucleic Acid Polymerase Chain Reaction.
HBV: Hepatitis B Virus.
HCV: Hepatitis C virus.
HIV: Human Immune Deficiency Virus.
HTC: HIV Counseling and Testing.
MP: Male Partner.
MPI: Male Partner Involvement.
MTCT: Mother to Child Transmission.
PMTCT: Prevention of Mother to Child Transmission.
WHO: World Health Organization.
