# Supplementary material for: Male partners involvement in human immune deficiency virus testing and counseling during prenatal care visits in Bichena town Westcentral Ethiopia: a cross-sectional study
Source: BMC Res Notes. 2022 Oct 20;15:327. doi: 10.1186/s13104-022-06215-9 (PMC9583477; doi:10.1186/s13104-022-06215-9)
Supplement: Supplementary file 1 — Supplementary Material 1 [file 13104_2022_6215_MOESM1_ESM.docx]

**Table S1:** Knowledge of MP about the services provided in ANC/PMTCT clinic in Bichena town, Westcentral, Ethiopia, 2019, (n=406).

| **Variables** | **Categories** | **Frequency** | **Percentage** |
| --- | --- | --- | --- |
| The services provided in ANC visits (multiple responses are possible) | Blood pressure measurement  Weight measurement  Abdominal examination  Blood test  Urine tests  Monitoring of fetal wellbeing  HIV counseling and testing  PMTCT  Iron and folic acid supplementation  Immunization against tetanus  Advice on danger signs of pregnancy  Advice on skilled birth attendance at birth  Advice on BPCRP  Advice on rest  Advice on dietary intake  Counsel on breastfeeding  Screening for sexually transmitted infections  Screening for hepatitis  Screening for diabetic Mellitus | 337  323  349  359  338  350  249  177  355  327  314  304  271  304  291  324  196  168  164 | 83.00  79.56  85.96  88.42  83.25  86.21  61.33  43.60  87.44  80.54  77.34  74.88  66.75  74.88  71.68  79.81  48.28  41.38  40.40 |
| Visiting ANC is important | Yes  No | 400  6 | 98.52  1.48 |
| Importance of the service provided at ANC visits (multiple responses are possible) | Access to skilled care  To prevent pregnancy complications.  Promote the health of the mother and fetus  To screen for HIV/AIDS | 397  314  381  338 | 97.79  77.34  93.84  83.25 |
| The appropriate time to start the ANC visit | Before 16 weeks  After 16 weeks | 268  138 | 66.00  34.00 |
| Recommended minimum number of ANC visits | 4^+^  <3 | 271  135 | 66.75  33.25 |
| A pregnant woman needs extra food | Yes  No | 394  12 | 97.04  2.96 |
| Alcohol consumption harmful to the fetus | Yes  No | 377  29 | 92.86  7.14 |
| Smoking harmful to the fetus | Yes  No | 388  18 | 95.57  4.43 |
| Who should accompany women for ANC visits | Husband  Others* | 238  168 | 58.62  41.38 |
| Where does a pregnant woman should give birth | Health facility  Home | 393  13 | 96.80  3.20 |
| Knowledge of male partner on ANC visits | Good knowledge  Poor knowledge | 267  139 | 65.77  34.23 |

*Wife alone, her family, husband's family, and relatives

**Table S2:** Health care facility and cultural influence on MPI in ANC/PMTCT at Bichena town, Westcentral, Ethiopia, 2019, (n=406)

| **Variables** | **Categories** | **Frequency** | **Percentage** |
| --- | --- | --- | --- |
| How long does it take to reach the nearest public health facility (one-way walking time) | Below 15  Minute 15-30  30 min and above | 58  244  104 | 14.29  60.10  25.61 |
| How was the attitude of the health care providers towards male partners (n=214) | Friendly  Unfriendly | 192  22 | 89.72  10.28 |
| Waiting time at ANC/PMTCT clinic (n=214) | Reasonable  Too long | 123  91 | 57.48  42.52 |
| Do you know maternal health service provision at the public health facility is free | Yes  No | 393  13 | 96.80  3.20 |
| Is the place where ANC/PMTCT service has given suitable for males (n=214) | Yes  No | 121  93 | 56.54  43.46 |
| Is the service provided at ANC is encouraged male involvement (n=214) | Yes  No | 183  31 | 85.51  14.49 |
| Can a pregnant woman visit ANC/PMTCT clinic without the permission of her partner in your community? | Yes  No | 386  20 | 95.07  4.93 |
| Who is selected to be the decision-maker to seek a health facility in your family | Together  Alone (husband or wife) | 355  51 | 87.44  12.56 |

**Figure S1:** Male partner involvement in ANC/PMTCT in Bichena town, Westcentral, Ethiopia, 2019, (n=406)
